# Identification of Body Behaviors and Facial Expressions Associated with Induced Orthopedic Pain in Four Equine Pain Scales

**DOI:** 10.3390/ani10112155

**Published:** 2020-11-19

**Authors:** Katrina Ask, Marie Rhodin, Lena-Mari Tamminen, Elin Hernlund, Pia Haubro Andersen

**Affiliations:** 1 Department of Anatomy, Physiology and Biochemistry, Swedish University of Agricultural Sciences, Box 7011, 75007 Uppsala, Sweden; marie.rhodin@slu.se (M.R.); elin.hernlund@uds.slu.se (E.H.); 2 Department of Clinical Sciences, Swedish University of Agricultural Sciences, Box 7054, 75007 Uppsala, Sweden; lena.mari.tamminen@slu.se (L.-M.T.); pia.haubro.andersen@slu.se (P.H.A.)

**Keywords:** horses, movement asymmetry, movement symmetry, lameness, reliability, pain predictor, pain indicator

## Abstract

**Simple Summary:**

Pain scales are tools developed to improve pain assessment in horses. They are based on behaviors and/or facial expressions, and the observer allocates a score based on the character of the behavior or facial expression. Little is known about behaviors and facial expressions at rest in horses with orthopedic pain since pain is mainly assessed by lameness evaluation during movement. The aim of this study was to describe how closely equine behaviors and facial expressions are associated with movement asymmetry and to identify combinations of behavior and expressions present in horses with induced orthopedic pain. Orthopedic pain was induced in eight horses and assessed in two ways; using four existing equine pain scales at rest, and by measuring movement asymmetry during movement. The association of behavior and facial expression items in the pain scales with actual lameness was analyzed. Posture-related behavior showed the strongest association, while facial expressions varied between horses. These results show that pain scales for orthopedic pain assessment would benefit from including posture, head position, location in the box stall, focus, interactive behavior, and facial expressions. This could improve orthopedic pain detection in horses during rest with mild lameness.

**Abstract:**

Equine orthopedic pain scales are targeted towards horses with moderate to severe orthopedic pain. Improved assessment of pain behavior and pain-related facial expressions at rest may refine orthopedic pain detection for mild lameness grades. Therefore, this study explored pain-related behaviors and facial expressions and sought to identify frequently occurring combinations. Orthopedic pain was induced by intra-articular LPS in eight horses, and objective movement asymmetry analyses were performed before and after induction together with pain assessments at rest. Three observers independently assessed horses in their box stalls, using four equine pain scales simultaneously. Increase in movement asymmetry after induction was used as a proxy for pain. Behaviors and facial expressions commonly co-occurred and were strongly associated with movement asymmetry. Posture-related scale items were the strongest predictors of movement asymmetry. Display of facial expressions at rest varied between horses but, when present, were strongly associated with movement asymmetry. Reliability of facial expression items was lower than reliability of behavioral items. These findings suggest that five body behaviors (posture, head position, location in the box stall, focus, and interactive behavior) should be included in a scale for live assessment of mild orthopedic pain. We also recommend inclusion of facial expressions in pain assessment.

## 1. Introduction

Pain scales are available as assessment tools for horses in pain and generally comprise composite-measure pain scales assessing pre-selected body behaviors and/or facial expressions. Body behaviors have been extensively studied and reviewed in horses for general pain or specific types of pain, such as orthopedic or visceral pain [1,2,3]. Facial expressions have been used for pain assessment in humans for many years, and have now been successfully introduced into pain assessment in horses, for acute pain types [4,5,6,7]. Behaviors and facial expressions are commonly seen together in horses experiencing pain [4], but can be difficult to assess. How well pain-related behaviors are expressed can depend on personality [8] and may be suppressed in response to an environment with possible threat [9]. Pain-related facial expressions can shift in presence as pain varies over time and can be influenced by the age of the animal, other affective states, or whether the pain is of an acute or chronic nature [10].

Scale reliability is important in clinical settings for scales to give reproducible results, independent of the observer. However, the level of reliability does not necessarily correlate to items that are good pain indicators. For lower degrees of pain, less distinct changes in behaviors or facial expressions may increase the variation in pain scores between observers, resulting in lower reliability. Good reliability obtained in studies with higher pain intensities should therefore not be generalized to studies with mild pain intensities, since the study groups are different [11]. So far, no study has evaluated reliability for scale items when low-degree orthopedic pain is assessed.

Current equine orthopedic pain scales are targeted towards horses with moderate to severe pain due to orthopedic surgery or laminitis [12,13,14]. Mild orthopedic pain of acute and chronic origin is mainly assessed subjectively, through evaluation of lameness grade during movement, and objectively, by kinetic or kinematic methods. It is commonly assumed that a higher lameness grade or movement asymmetry is equal to a higher degree of pain. However, this is not the case in human studies, where more complex relationships between pain and movement asymmetry are demonstrated. Both linear and non-linear positive relationships between biomechanical parameters (trunk asymmetry, vertebral motion, and range of motion in different joints) and pain have been shown [15,16,17], but also no relationship [18] or a negative relationship [19] between knee biomechanics and pain. Hence, a positive linear relationship between the magnitude of movement asymmetry or lameness and pain intensity should not be assumed. It may be assumed that horses at rest are in less pain, since they can decrease the load on the painful limb to a greater extent than is possible for horses in motion. The type of pain (acute or chronic) probably plays an important role in this regard. In acute pain, nociception occurs due to the inflammatory process and pain in an inflamed joint can be reduced by decreasing the load on the joint. This may result in reduced pain behaviors and facial expressions, suggesting that posture-related behaviors in acute orthopedic pain may be more stable than e.g., facial expressions. In chronic pain, central sensitization is often present, resulting in expansion of the painful area and an increase in pain intensity, which can be accompanied by stress, fatigue, and depression in humans [20,21]. Thus, decreased loading may not always be enough to alleviate pain, and horses at rest could then experience a high pain intensity, and display related pain behaviors and facial expressions. More research on pain behavior related to acute and chronic mild orthopedic pain in horses is needed to understand the relationship between different pain-related behaviors and facial expressions, and how they are affected by acute and chronic orthopedic pain. An improved assessment of pain behavior at rest could refine orthopedic pain detection.

Hence, for mild orthopedic pain, low-grade lameness during movement may be the only sign of pain observed and the lack of a gold standard for pain hampers determination of sensitivity and specificity for different scale items. In this study, we therefore used movement asymmetry as a proxy for orthopedic pain [11], assuming that increasing movement asymmetry post-induction was associated with presence of pain. The aim of this study was to investigate the relationship between scale items used in four equine pain scales and actual orthopedic pain. Specific objectives were to explore how well the scale item scores given at rest predicted lower degrees of movement asymmetry during movement, and to identify frequently occurring combinations of items in horses at rest with induced orthopedic pain. The reliability of each scale item was also evaluated. The first hypothesis tested was that a combination of behaviors and facial expressions is associated with movement asymmetry, since they commonly occur together during pain. We suspect that posture-related behaviors may reduce pain and other pain-related behaviors or facial expressions, why the second hypothesis tested was that assessment of body behaviors is more reliable than assessment of facial expressions.

## 2. Materials and Methods

The experimental protocol was approved by the Swedish Ethics Committee in accordance with the Swedish legislation on animal experiments (diary number 5.8.18-09822/2018). The study was designed to serve several purposes and the 3R’s were thoroughly considered designing the study. As few horses as possible were included and a fully reversible lameness induction model was used. It was important to induce lameness and let each horse be its own control, to achieve a standardized design and limit the variation in pain behavior between the horses.

### 2.1. Subjects

Lameness was inducted in six mares and two geldings (seven Standardbred trotters and one warmblood; mean ± SD age = 14.5 ± 3.7 years, mean ± SD body mass = 552 ± 39 kg and mean ± SD height at withers = 160 ± 2.78 cm). All horses were owned by the university or bought for/donated to the experiment. Before the experiment, the horses underwent a full clinical examination and subjective and objective lameness evaluations. All showed no signs of disease or >1 grade of lameness on a 0–5 lameness ordinal scale, where 0 = sound and 5 = non-weight bearing lameness. The 10–12 days immediately preceding lameness induction consisted of an acclimatization period with daily turnouts in a paddock, walker exercise, and handling and training. The horses were housed individually in box stalls with sawdust bedding. They were fed with hay three times a day and concentrate twice a day. The handling and training focused on positive reinforcement, to acclimatize the horses to palpation of the limbs, handling in different environments and lunging. The horses were all dewormed and hoof-trimmed during the first days of acclimatization.

### 2.2. Experimental Design and Induction of Orthopedic Pain

The last day of the acclimatization period contained an objective movement analysis to determine baseline movement asymmetry and what hindlimb to induce. The hindlimb with highest movement asymmetry was chosen for induction. Pain assessments in the box stall were also performed to determine baseline pain scores. One or two days later, mild to moderate orthopedic pain was induced early in the morning. After induction, the horse was taken back to its box stall for rest for 1.5 h, before the first pain assessment and movement measurement were performed. A minimum of three occasions with movement measurements and pain assessments were performed post-induction. Measurements were considered complete when each horse had returned to movement asymmetry similar to that of the baseline measurement.

Lipopolysaccharides (LPS) from E. coli O55:B5 (stock solution 1 mg/mL) were used to induce an acute inflammatory arthritis. Ready-made LPS solution (*L5418 Sigma*) was diluted with 0.9% sodium chloride to a final volume of 3 mL and a stock concentration of 1.167 ng/mL. The diluted solution was stored at −20 °C until the day of induction, when it was thawed and vortexed vigorously before intra-articular administration into the dorsomedial pouch of the tarsocrural joint. Routine aseptic techniques were used, where the horses were clipped and scrubbed on both hindlimbs. If a bandage or wound plaster was used after injection, it was added to the other hindlimb as well. A minimum of 3 mL synovia was extracted from the joint before LPS administration. If the induction resulted in a lameness grade >3 at trot, a protocol for rescue analgesia was initiated, comprising arthrocentesis and evacuation of synovia to reduce the intra-articular joint pressure and inflammatory pain.

### 2.3. Objective Movement Analysis

A movement measurement consisted of straight line walk and trot on hard and soft surface and lunging on soft surface. If movement asymmetry increased during the measurement, a second hard straight-line trot was performed. Each horse was equipped with seven skin-mounted spherical markers (38 mm diameter, Qualisys AB, Gothenburg, Sweden). Thirteen infrared optical motion capture cameras (Qualisys AB, Gothenburg, Sweden) recorded marker positions in 3D at 200 Hz and QTM software (version 2.11-2019.3, Qualisys AB, Gothenburg, Sweden) was used to track the positions of the markers. After visual inspection of tracking results, the data were exported and analyzed with custom-written scripts in MatLab [22]. Filtering was performed with a fourth-order zero-phase Butterworth filter, where the cut-off frequency was adjusted to the stride frequency of the horse [23]. Stride segmentation was based on peak detection of the vertical movement of the tubera sacrale, and left and right stride detection were performed using algorithms based on expected pelvic roll and yaw rotations of the tubera coxae [24]. Hard straight-line trot data from one marker placed over the poll and one marker placed between the tubera sacrale were included for further analysis. Vertical displacement asymmetry of the tubera sacrale is a well-established measure for hindlimb lameness [25], where pelvis reaches a lower position during the sound hindlimb stance. Vertical displacement asymmetry of the poll can be seen in some horses that reduce the weight on the lame hindlimb by shifting the weight forward, and the head and neck is lowered during the lame diagonal stance. Data from the other markers were collected for studies of more biomechanical focus. The difference between the two vertical displacement minima for each stride was calculated for head (HD_min_) and pelvis (PD_min_) in a way that assigned negative values to left-sided asymmetries. Mean total asymmetry for each measurement was computed as the sum of the absolute values for HD_min_/2 and PD_min_. The change in total movement asymmetry between baseline and induced measurements was then calculated and defined as total asymmetry score for each measurement. In addition, one or two experienced equine veterinarians subjectively graded the lameness during each movement measurement. An ordinal 0–5 lameness scale was used.

### 2.4. Pain Assessments 

Pain assessments were performed by direct observation at rest in the stable approximately 20 min before and 20 min after each movement asymmetry measurement. During this time, the horse was equipped/unequipped. For each pain assessment, three pain evaluators stood outside the box stall and performed simultaneous and independent live pain assessments on the same horse. Five pain evaluators took part in the study, two of whom (observers 1 and 2) were present for all assessments. Observers 3–5 changed between horses, based on availability. The observers consisted of three veterinarians, one agronomist, and one ethologist, and all had private or professional equestrian experience. Prior to pain assessments, the evaluators familiarised themselves thoroughly with the pain scales, using published available score sheets, descriptions, and scientific reports. Only observer 1 participated during the induction, while the other observers only saw the horses in their box stalls during pain assessment. They were therefore blinded to the limb of induction and the increase in movement asymmetry post-induction.

Four equine pain scales were used, in the following order: Horse Grimace Scale (HGS) [4], Equine Utrecht University Scale of Facial Assessment of Pain (EQUUS-FAP) [5,6,14], Equine Pain Scale (EPS) [26] and Composite Pain Scale (CPS) [12]. The HGS and EQUUS-FAP scales primarily assess facial expressions and have been used previously to assess orthopedic pain of moderate to a severe degree. EPS assesses body behavior and presence of pain face and has been recommended for general pain. CPS assesses body behavior and physiological parameters and has been used for assessment of post-surgical orthopedic pain. The scales contain six to 13 items (see Supplementary Materials, Table S1) and item scores range from 0 to 2 for HGS and EQUUS-FAP to 0–4 for EPS and 0–3 for CPS. The EQUUS-FAP, EPS, and CPS scales are designed for direct (live) scoring, while HGS is designed for indirect (video) scoring [4,13]. The pain assessments with HGS, EQUUS-FAP, and EPS lasted for two minutes each. The assessments with CPS lasted for five minutes, where the last two minutes consisted of measuring physiological parameters and palpation of the limbs.

### 2.5. Statistics

Statistical computations and analyses were executed in R [27]. Data from movement measurements performed until each horse reached the maximum total asymmetry score, and associated pain assessments, were included for further analyses. The highest increase in movement asymmetry was determined manually for each individual. Pain assessments performed before and after selected movement measurements were included. Mean and standard deviation were computed for item scores and movement asymmetry data. Reliability of each scale item was analyzed with Kendall’s coefficient of concordance (*W*) [28] for agreement of ordinal ranking data [29]. Physiological parameters of CPS were excluded for reliability testing since they were objectively measured. The role of scale items in predicting movement asymmetry was explored with Lasso regression models, that were fitted using the package ‘glmnet’ [30] (alpha = 1), to identify the combination of predictors associated with total asymmetry score for each scale and for all scales in the dataset. The models were fitted with 10-fold cross-validation. Scale items were analyzed as factors, while horse and observer were included as fixed effects to account for individual variation. Scores from pain assessments pre- and post-movement measurements were included. When there were two straight-line trot measurements, both were included and the first associated with the pre-pain assessment and the second with the post-pain assessment. The lambda generating models with the minimum mean cross-validated error was selected. To further assess the associations between scale items from different scales, multiple correspondence analysis (MCA) was performed using the package ‘FactoMineR’ [31]. The 33 largest dimensions (explaining a minimum of 1% of the variation within scale items) were then included in a linear regression with movement asymmetry as the outcome. The model was reduced using Akaike information criterion (AIC) and the components of the significant (*p* < 0.01) dimensions in the final model were interpreted. Horse and observer were included as random effects and the distribution of the residuals was controlled for signs of temporal autocorrelation in the final model. Results were plotted using ‘ggplot2′ [32].

## 3. Results

**Figure 1:**
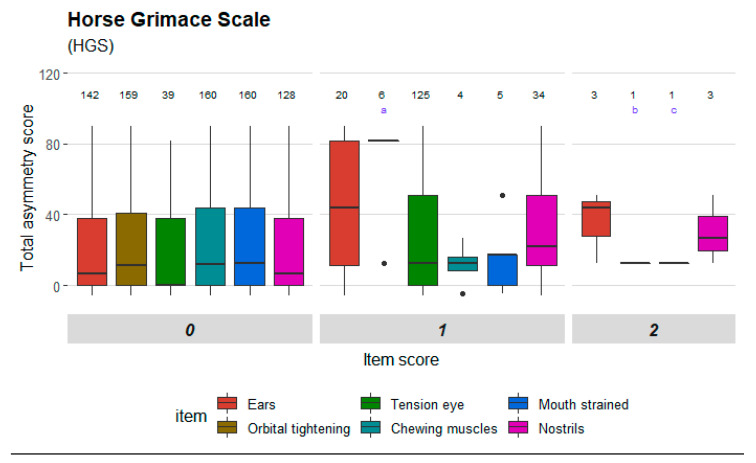
Distribution of item scores for Horse Grimace Scale (HGS). Total asymmetry score is presented on the y-axis. Scale items are presented on the x-axis and divided into the item scores given (ranging from 0 to 2). Item scores for all observers are included and number of scores (*n*) is stated above each box. Outliers are included and shown as black dots. The black line in the boxes shows the median and the upper and lower ends of the boxes show the upper and lower quartile. The upper and lower whiskers show the highest and lowest 25% of the data. Some of the boxes contain few observations and low spread, and appear as horizontal lines where the color is not visible. Instead, they are marked with letters in the diagram: a—orbital tightening, b—tension above the eye area, **c**—prominent strained chewing muscles.

**Figure 2:**
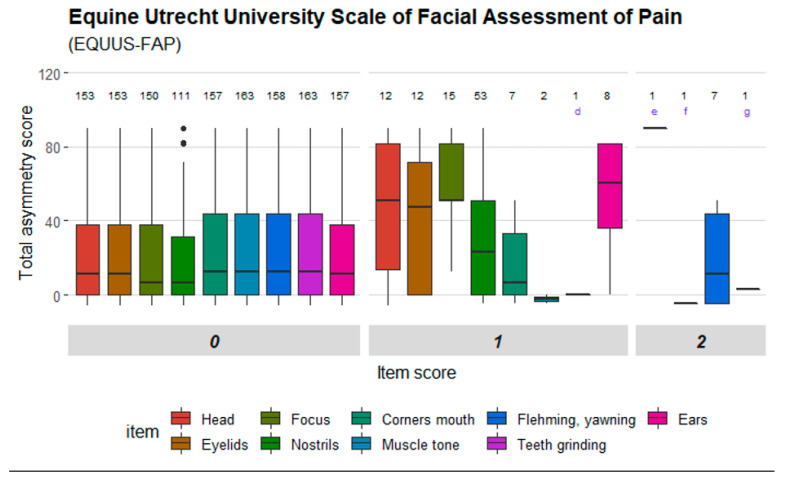
Distribution of item scores for Equine Utrecht University Scale of Facial Assessment of Pain (EQUUS-FAP). Total asymmetry score is presented on the y-axis. Scale items are presented on the x-axis and divided into the item scores given (ranging from 0 to 2). Letters in the diagram: d—teeth grinding/moaning, e—nostrils, f—corners of mouth/lips, g—teeth grinding/moaning.

**Figure 3:**
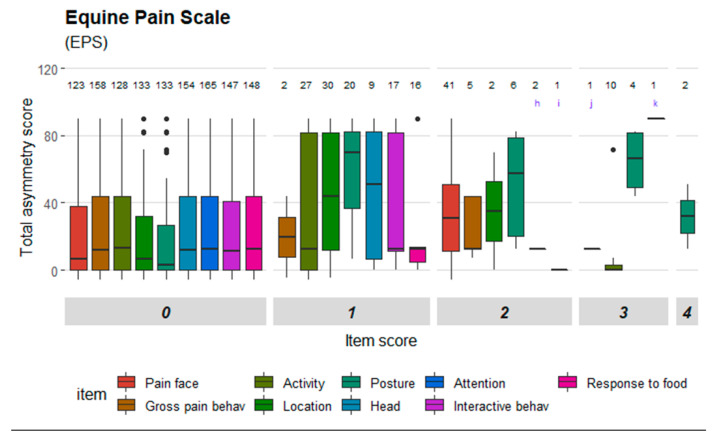
Distribution of item scores for Equine Pain Scale (EPS). Total asymmetry score is presented on the y-axis. Scale items are presented on the x-axis and divided into the item scores given (ranging from 0 to 4). Letters in the diagram: h—head position, i—interactive behavior, j—pain face, k—response to food.

**Figure 4:**
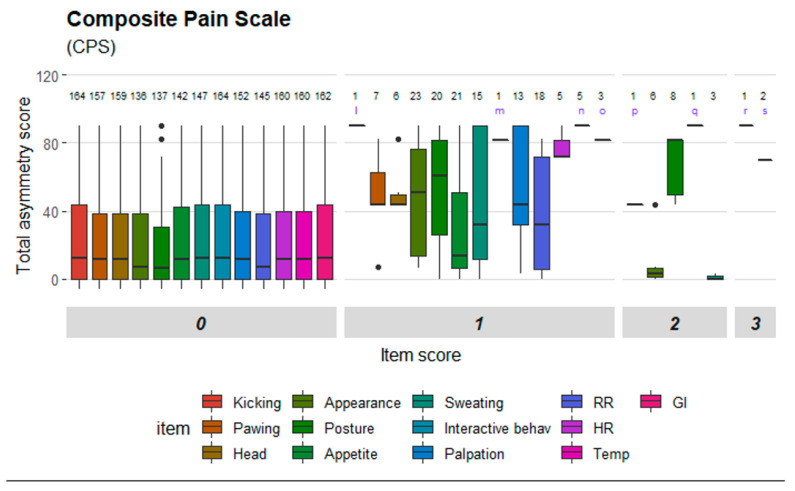
Distribution of item scores for Composite Pain Scale (CPS). Total asymmetry score is presented on the y-axis. Scale items are presented on the x-axis and divided into the item scores given (ranging from 0 to 3). Letters in the diagram: l— kicking abdomen, m—interactive behavior, n—rectal temperature, o—digestive sounds, p—pawing on the floor, q—appetite, r—appetite, s—respiratory rate.

Mean (±SD) number of movement measurements per horse was 4.25 (±1.04) and mean number of pain assessments was 6.88 (±1.46). Three left and five right hindlimbs were induced and mean (±SD) maximum increase in total asymmetry score was 61 mm (±24 mm). Mean (± SD) PD_min_ value was 3 mm (±3 mm) for baseline measurements, and 46 mm (±20 mm) for measurements where the maximum movement asymmetry was reached. The baseline subjective lameness score was 0 for all horses, except two that had 0.25 and 0.5 grades respectively. The maximum subject lameness score varied between 2 and 4 grades (mean 2.94 and SD 0.78). Changes in total asymmetry score and PD_min_ can be seen in detail in Supplementary Materials File S2. Rescue analgesia (reducing synovial volume by arthrocentesis) was performed in two horses. A total of 37 scale items were included in the dataset, some of which were similar despite originating from different scales. Mean (±SD) item scores before and after induction are presented in Supplementary Materials Table S1. Figure 1, Figure 2, Figure 3 and Figure 4 illustrate the distribution of item scores and that item scores of zero were present for all degrees of movement asymmetry. However, certain items stood out and only achieved scores above zero when movement asymmetry increased. This was seen especially for ‘ears’ and ‘nostrils’ in HGS (Figure 1); ‘head’, ‘focus’ and ‘ears’ in EQUUS-FAP (Figure 2); ‘location’, ‘posture’, ‘pain face’, ‘gross pain behavior’ and ‘head’ in EPS (Figure 3); and ‘pawing’, ‘head’, ‘appearance’, ‘posture’ and ‘response to palpation’ in CPS (Figure 4). Physiological parameters in CPS had also scores >0 when movement asymmetry increased (Figure 4).

Agreement between observers was considered very strong for W > 0.9, strong for W 0.7–0.9, moderate for W 0.5–0.7, and weak or very weak for W < 0.5 [33]. One scale item in HGS showed strong agreement (‘orbital tightening’), while two scale items showed moderate agreement and three poor agreement. Two scale items in EQUUS-FAP showed strong agreement (‘focus’ and ‘flehmen and/or yawning’) and seven items had weak agreement. Five items in EPS had strong agreement (‘gross pain behavior’, ‘activity’, ‘posture/weight bearing’, ‘interactive behavior’ and ‘response to food’), one item showed moderate agreement and three items weak agreement. ‘Sweating’ in CPS was the only item with very strong agreement, and four items in CPS had strong agreement (‘pawing’, ‘posture’, ‘appetite’, ‘response to palpation’). Two items had moderate agreement and two items had weak agreement. The coefficients are presented in Table 1.

**Figure 5:**
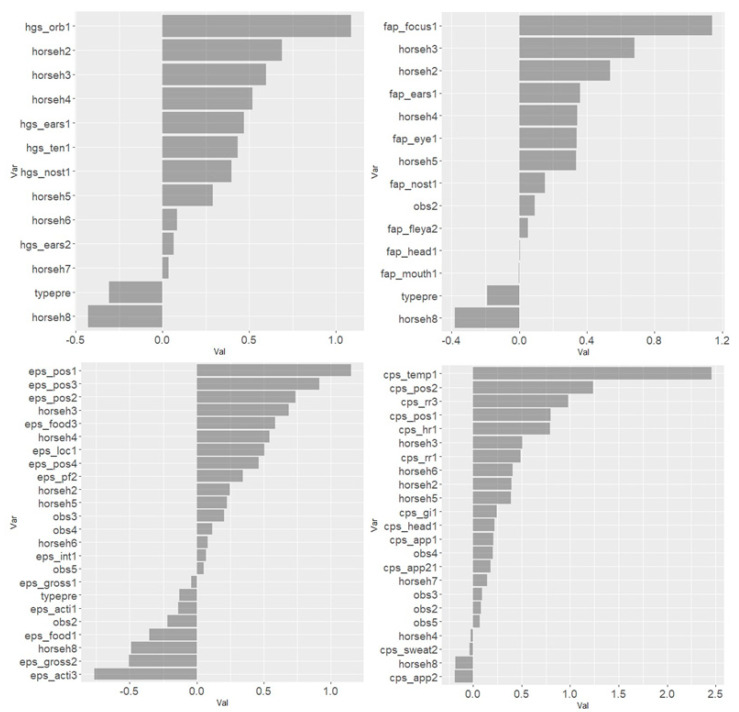
Coefficient of Lasso regression models with lambdas generating the minimum mean cross-validated error when using scale items to predict movement asymmetry. Scale items from the four equine pain scales (Horse Grimace Scale (HGS), Equine Utrecht University Scale of Facial Assessment of Pain (EQUUS-FAP), Equine Pain Scale (EPS) and Composite Pain Scale (CPS)) were analyzed in separate models. Scale items with a positive coefficient (to the right) were positively associated with total asymmetry score, used here as a proxy for orthopedic pain. The number after the scale item is the item score, for example hgs_orb1 means an item score of 1 for orbital tightening in HGS.

**Figure 6:**
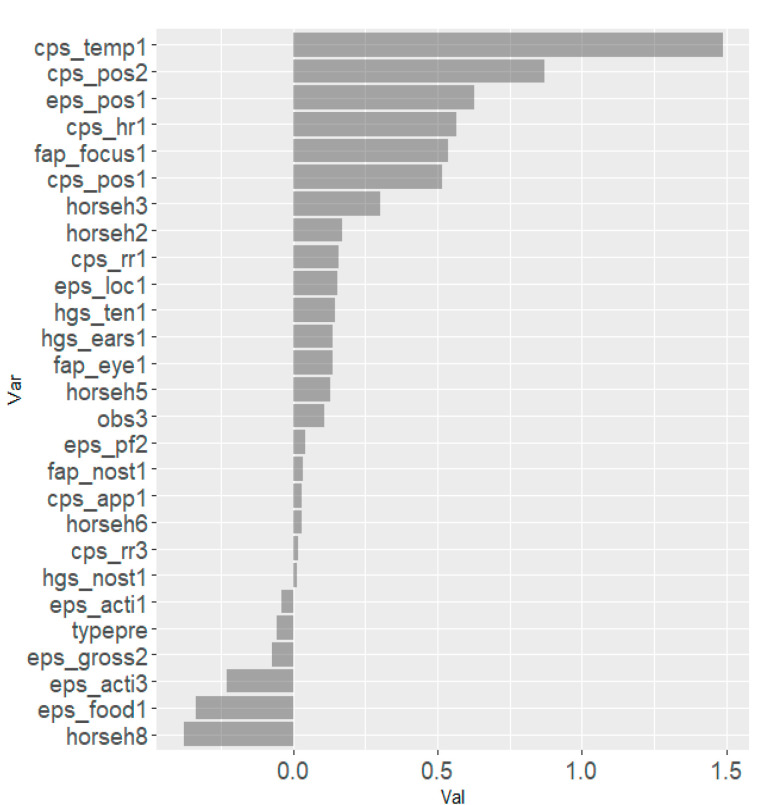
Coefficient of Lasso regression models with lambdas generating the minimum mean cross-validated error when using scale items of all scales to predict movement asymmetry. Scale items from the four equine pain scales (Horse Grimace Scale (HGS), Equine Utrecht University Scale of Facial Assessment of Pain (EQUUS-FAP), Equine Pain Scale (EPS) and Composite Pain Scale (CPS)) were analyzed in the same model.

The results from the Lasso regression indicated that the combination of following scale items within each scale were most strongly associated with the total asymmetry score: ‘orbital tightening’ for HGS, ‘focus’ for EQUUS-FAP, ‘posture’ for EPS, and ‘temperature’ and ‘posture’ for CPS (Figure 5). For all scales, horses h1–h8 seemed to be relatively strongly associated with total asymmetry score and horse h4 in particular had a strong association on three scales. The effects of horse and observer were accounted for in scale items associated with total asymmetry score. Scale items negatively associated with total asymmetry score were ‘teeth grinding’ (EQUUS-FAP), ‘gross pain behavior’ (EPS) and ‘sweating’ (CPS). The model best describing changes in movement asymmetry with all scale items included was ‘posture’ in CPS and EPS, together with ‘temperature’ and ‘heart rate’ in CPS, and ‘focus’ in EQUUS-FAP (Figure 6).

**Figure 7:**
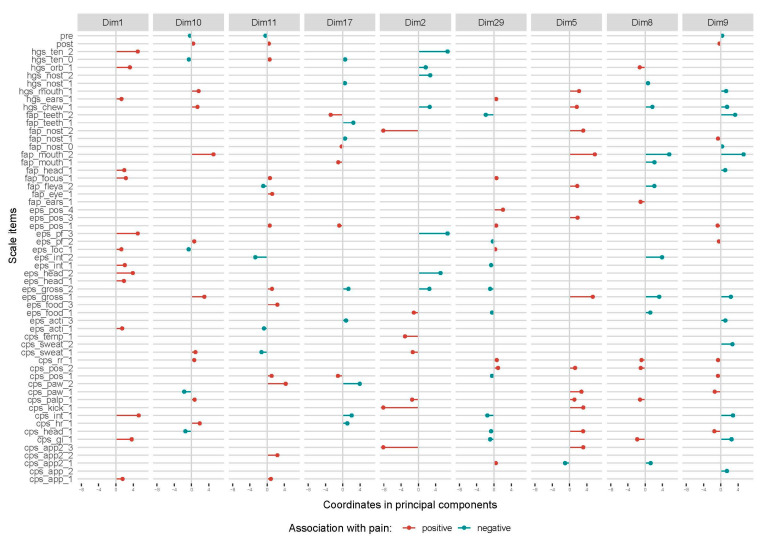
Results of multiple component analysis (MCA) illustrating the most significant dimensions. All scale items are included. The variance explained by each dimension is stated in the diagram. Items with red markers have a positive association with total asymmetry score, while items with blue markers have a negative association.

Thirty-three MCA-dimensions explaining from 10% to 1% of the variation in the data were included in the linear regression. Nine dimensions were significantly associated with movement asymmetry (Table 2) and the composition of these dimensions is presented in Figure 7. Facial expressions and ‘pain face’ were seen together with ‘interaction’ and ‘activity’ parameters in horses with increased total asymmetry score in dimension 1, with ‘gross pain behavior’ and ‘postural changes’ in dimension 5, and ‘gross pain behavior’ in dimension 10. Dimension 29 was most strongly associated with movement asymmetry and involved an interesting combination of behaviors (‘stiffly backwards ears’, ‘focus’, ‘posture’, ‘location’ and ‘appetite’) and lack of pain-related facial expressions in horses with increased total asymmetry score. ‘Interaction’, ‘gross pain behavior’ and ‘head position’ were other behaviors not seen in horses in this dimension. A similar pattern was seen for dimension 8 and 9, where many facial expressions and ‘gross pain behavior’ had a negative association with movement asymmetry, while ‘posture’ and ‘pawing’ had a positive association. In dimension 2, facial expressions and ‘pain face’ were not seen in horses with increased total asymmetry score if they showed gross pain behaviors, were kicking, had lowered head and decreased appetite, and had increased temperature.

## 4. Discussion

The scale item most strongly associated with movement asymmetry when comparing all scale items in a Lasso regression model was ‘body temperature’ from CPS, closely followed by ‘posture’ (EPS and CPS) and ‘heart rate’ (CPS). Since movement asymmetry was induced by intra-articular administration of LPS, which is a pyrogen, it is not surprising that increased body temperature was a strong predictor of pain [34,35]. The association between heart rate and orthopedic pain is not consistent in studies [12,36], however heart rate is closely associated with body temperature [37,38]. This may indicate that increased heart rate in our subjects was associated with increased body temperature. The items ‘focus’ and ‘flehmen and yawning’ from EQUUS-FAP were also strong predictors. Facial expressions were in general not as strong pain predictors as behaviors. ‘Orbital tightening’ from HGS, and ‘eyelids’ and ‘ears’ from EQUUS-FAP had larger coefficients than other facial expressions in this study, but ‘stiffly backwards ears’, ‘tension around the eye area’, ‘nostrils’ and ‘pain face’ were also positively associated with pain. As the Lasso tends to select one variable in case of correlated variables, this indicates that separate expressions are important in themselves and that a combination of facial expressions indicates increased pain.

When association between scale items and pain was compared within each scale, ‘posture’ had large coefficients in both scales assessing posture (EPS and CPS). ‘Tension around the eye area’ and‘stiffly backwards ears’ from HGS, and ‘eyelids’ from EQUUS-FAP were the facial expressions with the largest coefficient, i.e., they were important variables for predicting change in movement asymmetry. Interestingly, ‘gross pain behavior’ from EPS was negatively associated with pain in this study, which contradicts published research results [26,36,39]. Behaviors included in ‘gross pain behavior’ in EPS are yawning, mouth playing, flehmen, stretching, kicking abdomen, tail swishing and sweating, where ‘flehmen and/or yawning’ from EQUUS-FAP was positively associated with pain. It is possible that some behaviors included in ‘gross pain behavior’ were present due to reasons other than pain, for instance emotional stress. When inspecting the scores given for ‘gross pain behavior’ in this study, three horses (h2, h3 and h8) had positive scores post-induction, but not during assumed maximum pain level, when total asymmetry score peaked. Since the frequency of positive scores was low, this can affect the statistical outcome and result in a negative prediction.

Interestingly, some of the individual horses’ large coefficients in the Lasso regressions (Figure 5), are underlying the importance of individual characteristics when assessing pain. Theoretically, this is not surprising. Since pain is an experience and related to the *personality* of the horse [8], all horses cannot be expected to show the same frequency and intensity in pain behaviors, as is also the case for humans [40]. This adds to the limitations with small sample sizes in pain studies, where larger samples could have compensated for large individual variations.

Scale items occurring together varied greatly but, in general, body behaviors and facial expressions were seen together in horses experiencing pain. Facial expressions were positively associated with movement asymmetry in several of the dimensions derived from the MCA analysis, indicating that they are important indicators of pain. However, no dimension contained all facial expressions included in a pain face. Eye- and ear-related facial expressions were found in one dimension, and lower facial expressions in another. This is consistent with results from the Lasso regression indicating that facial expressions are not always correlated but add value individually. Four different combinations of facial expressions are reported to be present during pain in humans, some more stable than others [41], illustrating individual variations in how a pain face is expressed. The results in the present study may indicate similar variations in horses. In humans, upper facial expressions, such as brow lowering and nose wrinkling, are of more importance when assessing pain [42], and it is possible that the observer may subconsciously see these features more easily in horses but overlook other relevant facial expressions. Studies of facial expressions of pain in humans describe complex relationships between facial expressions and social context, with an unsafe environment or the presence of strangers sometimes seeming to decrease facial expressions, even during high pain intensity [43,44]. Whether such explanations are also valid for horses needs to be investigated further, for example by comparison of facial expressions with and without observers present. So far, discomfort behavior in general seem to decrease when caretaking staff are approaching equine patients [45]. We performed all pain assessments live, with three unknown observers present. Behaviors of an interactive character were seen together with upper facial expressions, while postural changes were seen with lower facial expressions (Figure 7). Lower facial expressions were also seen with gross pain behavior and may represent different pain intensities, since gross pain behavior is indicative of higher pain intensity. Facial expressions were not always present together with postural changes, even though total movement asymmetry was high. As discussed in the introduction, lame horses can be expected to modify their pain at rest, by simply avoiding situations that may increase pain intensity, for example loading of the painful limb. This may result in other behaviors occurring less frequently during pain [46,47]. The patterns seen in our MCA may confirm this theory since facial expressions rarely were seen when only posture-behaviors were present. Lowered head was also a behavior present in several dimensions and may indicate a depressed clinical state, a behavior often seen together with different pain intensities or in horses with pain for long duration and sleep deprivation [3]. Whether a depressive state is present in horses needs to be investigated for both acute and chronic pain. Scale items with few positive scores, such as ‘temperature’, ‘sweating’, ‘response to palpation’, ‘kicking abdomen’ and ‘appetite’, were clustered together. Behavioral changes in these items often indicate high pain intensity, but were seen in horses without pain face or gross pain behavior. However, little weight can be given to this clustering, due to the few positive scores.

Reliability was estimated for each scale item and surprisingly low agreement was found for three independent observers scoring all items from all scales. Low agreement may indicate difficulties in interpreting the scale items and/or difficulties in seeing what to score, leading to larger variance in the scores. If there were difficulties in seeing a facial expression, it can be argued that scale items assessing this expression still have the same level of reliability. Our results showed that for instance ‘stiffly backward ears’ from HGS and ‘ears’ from EQUUS-FAP have moderate vs. low agreement. This suggests that the item ‘stiffly backwards ears’ is easier to interpret than the item ‘ears’. The same phenomenon was seen for some behavior items such as ‘head position’ in EPS, ‘head’ in EQUUS-FAP and ‘head movement’ in CPS, where low agreement was seen for items of EPS and EQUUS-FAP, while the item of CPS had moderate agreement. This may indicate and that more extensive introduction and training are needed to be able to interpret the items correctly [48] In addition, the long scoring sessions may have contributed to observer fatigue [49]. Nevertheless, there is a reason to believe that the pain scales may need improvement of the scale item definitions to be more user-friendly and to increase the reliability. The generally low reliability may affect the results in this study, and an important bias is that easily detected behaviors may have achieved higher scores compared to behaviors or facial expressions being harder to identify.

Inter-observer agreement for facial expressions was lower than that for body behaviors, which is an important finding for pain assessment quality in horses. It can be due to the scale limitations above, but it can also be due to other factors such as facial expressions being harder to identify for the human eye. It has been argued that humans have an innate tendency to focus on the face region, and that this could facilitate the use of facial expressions in monitoring welfare in rabbits [50]. This is apparently not the case for the species in this study. A possible bias may be the potential influence of observing different scale items, for example, lameness or gross pain behavior, when scoring other more difficult or subtle scale items. For instance, a facial expression may be scored differently depending on whether the observer has seen gross pain behavior or not. More analyses of the material are needed to investigate this potential bias effect and advice on whether facial expressions and body behaviors should be evaluated blinded to each other, or not. An option could be automated scoring of facial expressions from video or the application of more objective coding systems such as EquiFACS [51].

Of the 37 scale items evaluated in this study, several were included in more than one pain scale, but were weighted differently in the scale design. How items should be weighted may differ between pain intensities, since behaviors that are good indicators of pain at higher pain intensities are not necessarily good indicators at lower pain intensities. This could be overcome for instance by specifying the pain intensity for which a pain scale is designed or by introducing cut-off values for different pain intensities. The results of this study indicate that posture should be weighted higher than other behaviors for mild orthopedic pain at rest. It is however important to emphasize that only hindlimb lameness was induced in this study and that posture-related changes may show higher or lower associations with movement asymmetry if the lameness is located in the front limb. Whether the localization of the lameness should be included in an orthopedic pain scale cannot be determined from this study. Facial expressions seem to be of less value during rest, due to the variation observed together with postural changes.

## 5. Conclusions

This study showed that pain scale items related to posture at rest were the strongest behavioral predictors of movement asymmetry in horses with mild orthopedic pain. Behaviors and facial expressions commonly occurred together and were strongly associated with movement asymmetry. The study also showed that the presence of facial expressions at rest can vary in horses with low-grade lameness but, when present, facial expressions were strongly associated with movement asymmetry. Reliability was lower for facial expression items than for behavioral items, indicating that it can be difficult to assess facial expressions by direct observation.

The results obtained suggest that pain scales combining facial expressions with body behaviors should be used when performing direct pain assessment of horses with mild orthopedic pain at rest. We propose that five body behaviors (posture, head position, location in the box stall, focus, and interactive behavior) be included in an optimal scale for live assessment of mild orthopedic pain. We also propose that the posture item be refined to include more levels. We recommend that facial expressions during pain assessment of mild orthopedic pain at rest should be considered together with other behaviors and that observers may need more extensive training to be able to assess them.

## Figures and Tables

**Table 1:** Inter-observer agreement estimated with Kendall’s coefficient of concordance (*W*) for the four pain scales tested. Pain scores for each scale item and three observers are included.

Scale	Scale Item	*W*	*p*-Value
Horse Grimace Scale (HGS)	Stiffly backward ears (ears)	0.567	<0.001 ***
	Orbital tightening (orb)	0.794	<0.001 ***
	Tension above the eye area (ten)	0.470	0.025 *
	Prominent strained chewing muscles (chew)	0.421	0.091
	Mouth strained and pronounced chin (mouth)	0.418	0.099
	Strained nostrils and flattening of the profile (nost)	0.575	<0.001 ***
Equine Utrecht University Scale of Facial Assessment of Pain (EQUUS-FAP)	Head (head)	0.383	<0.001 ***
	Eyelids (eye)	0.433	0.068
	Focus (focus)	0.819	<0.001 ***
	Nostrils (nost)	0.405	0.134
	Corners mouth/lips (mouth)	0.316	0.584
	Muscle tone head (tone)	0.329	0.500
	Flehmen and/or yawning (fleya)	0.751	<0.001 ***
	Teeth grinding and/or moaning (teeth)	0.333	0.474
	Ears (ears)	0.376	0.242
Equine Pain Scale (EPS)	Pain face (pf)	0.428	0.078
	Gross pain behavior (gross)	0.753	<0.001 ***
	Activity (act)	0.722	<0.001 ***
	Location in the stall (loc)	0.605	<0.001 ***
	Posture/weight bearing (pos)	0.743	<0.001 ***
	Head position (head)	0.409	0.122
	Attention towards painful area (att)	0.333	0.474
	Interactive behavior (int)	0.775	<0.001 ***
	Response to food (food)	0.881	<0.001 ***
Composite Pain Scale (CPS)	Kicking abdomen (kick)	0.333	0.474
	Pawing on the floor (paw)	0.763	<0.001 ***
	Head movement (head)	0.538	<0.003 **
	Appearance (app)	0.646	<0.001 ***
	Posture (pos)	0.741	<0.001 ***
	Appetite (app2)	0.837	<0.001 ***
	Sweating (sweat)	0.920	<0.001 ***
	Interactive behavior (int)	0.333	0.474
	Response to palpation of painful area (palp)	0.844	<0.001 ***

Significant coefficients are indicated as: ns = *p* > 0.05, * *p* < 0.05, ** *p* < 0.01, *** *p* < 0.001. Abbreviations used for statistical analysis are stated in brackets for each item. Only behavioral items in CPS are included.

**Table 2:** Values of significant dimensions in multiple component analysis (MCA).

Dimension	Beta	SE	z-Value	*p*-Value
1	32.08	3.80	8.44	<0.001 ***
9	−28.66	6.30	−4.55	<0.001 ***
29	44.59	11.54	3.86	<0.001 ***
10	24.69	7.10	3.48	<0.001 ***
8	−21.84	6.39	−3.42	<0.001 ***
2	−16.97	5.32	−3.19	0.00141 **
5	17.28	5.87	2.94	0.00324 **
17	−24.34	8.62	−2.82	0.00477 **
11	19.42	7.54	2.58	0.01000 *

Significant coefficients are indicated as: ns = *p* > 0.05, * *p* < 0.05, ** *p* < 0.01, *** *p* < 0.001. SE = standard error of the mean. Variance of random effects: horse 0.25, observer 4.2 × 10^−8^.

## Supplementary Materials

The following are available online at https://www.mdpi.com/2076-2615/10/11/2155/s1, Table S1: Mean (± SD) scores for each scale item, File S2: Dataset in an Excel book used for data analysis.
