# Supplementary material for: Identification of Body Behaviors and Facial Expressions Associated with Induced Orthopedic Pain in Four Equine Pain Scales
Source: Animals (Basel). 2020 Nov 19;10(11):2155. doi: 10.3390/ani10112155 (PMC7699379; doi:10.3390/ani10112155)
Supplement: Supplementary file 1 [file animals-10-02155-s001.zip › Table S1.pdf]

Table S1. *Mean (± SD) scores for each scale item before and after lameness induction. Pain assessments performed up to each horse reached the maximum total asymmetry score are included. Number of pain assessments (n) are stated in the table. Scores from all observers are included. SD = standard deviation of the mean.*

**Scale**	**Scale item**	**Before induction** *(n = 16)*		**After induction** *(n = 39)*	
		Mean	*(± SD)*	*Mean*	*(± SD)*
**Horse Grimace Scale** (HGS)	Stiffly backward ears	0.063	0.245	0.197	0.459
	Orbital tightening	0.000	0.000	0.051	0.222
	Tension above the eye area	0.625	0.489	0.829	0.400
	Prominent strained chewing muscles	0.000	0.000	0.051	0.258
	Mouth strained and pronounced chin	0.021	0.144	0.034	0.182
	Strained nostrils and flattening of the profile	0.083	0.279	0.308	0.516
**Equine Utrecht University Scale of Facial Assessment of Pain** (EQUUS-FAP)	Head	0.000	0.000	0.103	0.305
	Eyelids	0.063	0.245	0.077	0.268
	Focus	0.000	0.000	0.128	0.336
	Nostrils	0.292	0.459	0.350	0.497
	Corners mouth/lips	0.042	0.202	0.060	0.272
	Muscle tone head	0.021	0.144	0.009	0.092
	Flehmen and/or yawning	0.000	0.000	0.120	0.476
	Teeth grinding and/or moaning	0.021	0.144	0.017	0.185
	Ears	0.021	0.144	0.060	0.238
**Equine Pain Scale** (EPS)	Pain face	0.167	0.559	0.658	0.957
	Gross pain behaviour	0.000	0.000	0.103	0.423
	Activity (act)	0.500	1.011	0.282	0.641
	Location in the stall	0.125	0.393	0.239	0.448
	Posture/weight bearing	0.000	0.000	0.444	0.875
	Head position	0.042	0.202	0.094	0.347
	Attention towards painful area	0.000	0.000	0.000	0.000
	Interactive behaviour	0.125	0.393	0.111	0.316
	Response to food	0.083	0.279	0.128	0.406
**Composite Pain Scale** (CPS)	Kicking abdomen	0.000	0.000	0.009	0.092
	Pawing on the floor	0.000	0.000	0.077	0.298
	Head movement	0.000	0.000	0.051	0.222
	Appearance	0.083	0.404	0.265	0.515
	Posture	0.021	0.144	0.299	0.591
	Appetite	0.104	0.309	0.179	0.466
	Sweating	0.104	0.425	0.137	0.369
	Interactive behaviour	0.000	0.000	0.009	0.092
	Response to palpation of painful area	0.000	0.000	0.111	0.316
	Respiratory rate	0.042	0.202	0.188	0.507
	Heart rate	0.000	0.000	0.043	0.203
	Rectal temperature	0.000	0.000	0.043	0.203
	Digestive sounds	0.000	0.000	0.026	0.159
